# Prognostic value of combined inflammatory and nutritional biomarkers in HCC within the Milan criteria after hepatectomy

**DOI:** 10.3389/fonc.2022.947302

**Published:** 2022-09-05

**Authors:** Hanxin Feng, Feng Xu, Yang Zhao, Tianqiang Jin, Jianbo Liu, Rui Li, Tianyi Zhou, Chaoliu Dai

**Affiliations:** Department of General Surgery, Shengjing Hospital of China Medical University, Shenyang, China

**Keywords:** hepatocellular carcinoma, Milan criteria, inflammation, prognostic nutritional index, prognostic factor

## Abstract

**Aims:**

This study aimed to evaluate the predictive value of the combined prognostic nutritional index (PNI) and GGT/ALT for the postoperative prognosis of patients with hepatocellular carcinoma (HCC) within Milan criteria undergoing radical hepatectomy.

**Methods:**

This single-center retrospective study included 283 patients with HCC within the Milan criteria who underwent hepatectomy. The receiver operating characteristic (ROC) curve was used to calculate the optimal PNI and GGT/ALT cut-off values. Pre-treatment PNI, GGT/ALT, and PNI-GGT/ALT grades were calculated. Overall survival (OS) and recurrence-free survival (RFS) were estimated using the Kaplan–Meier method, and multivariate analysis was used to identify prognostic factors.

**Results:**

Multivariate Cox regression analysis identified that the PNI, GGT/ALT, tumor number were significant prognostic markers for OS, and that the GGT/ALT, tumor number were significant prognostic markers for OS. The survival curves showed that low PNI, high GGT/ALT ratio, and high PNI-GGT/ALT grade were associated with poorer OS and DFS. With an area under the curve (AUC) of 0.690, PNI-GGT/ALT outperformed each individual score.

**Conclusion:**

PNI-GGT/ALT, a new prognostic scoring model, qualifies as a novel prognostic predictor for patients with HCC within the Milan criteria after curative resection.

## Introduction

Hepatocellular carcinoma (HCC) is the sixth most common type of malignant tumor, ranked as the fifth most common in men and ninth in women (1). Though hepatic resection has been recommended as the first choice for curative treatments, only approximately 15%–20% of patients are deemed suitable candidates for surgical resection at HCC diagnosis (2). Even after hepatectomy, long-term prognosis remains unsatisfactory with an estimated 5-year overall survival (OS) rate of 50% (3). Therefore, a more accurate estimate of the tumor burden and prognosis of patients has become a concern for HCC management.

Cancer-related inflammation is the seventh hallmark of cancer (4). HCC is a typical inflammation-driven disease that primarily develops due to underlying chronic liver inflammation; chronic HBV and HCV infection account for 80% of HCC cases globally (5). Inflammation contributes to the development and progression of HCC by supporting cancer stem cells, promoting proliferative and survival signaling, evading immune surveillance, inducing angiogenesis, activating invasion, metastasis, and inducing genomic instability (6).

The prognostic nutritional index (PNI), which is obtained through serum albumin (ALB) and lymphocyte count in the peripheral blood, is a simple and practical indicator of systemic inflammatory response to assess perioperative immune and nutritional status (7). PNI was initially proposed to predict the prognosis of patients with gastrointestinal malignancies (8), and is a good prognostic indicator for prostate, pancreatic, lung, and gallbladder cancers (9–12). Recent evidence demonstrates that patients with HCC with a lower preoperative PNI have a worse prognosis (13, 14).

Both gamma-glutamyl transferase (GGT) and alanine aminotransferase (ALT) are well-established hepatitis parameters that reflect inflammatory disturbances in the liver. A lower GGT/ALT ratio was initially proposed to be prognostically associated with better condition and treatment response in viral hepatitis (15, 16). Furthermore, the analysis of the GGT/ALT ratio was valuable for the diagnosis of HCC (17). Ju et al. reported that GGT/ALT was a predictor for the prognosis of Child-Pugh A HCC resections, a high GGT/ALT ratio was associated with poor prognosis of patients with HCC after liver resection (18). Zhang et al. showed that a combination of PNI and GGT/ALT may be a useful prognostic predictor in patients with HBV-associated HCC, even better than the tumor-node-metastasis (TNM) stage (19).

Hence, this study aimed to evaluate the prognostic value of PNI and GGT/ALT in HCC within the Milan criteria and investigate whether preoperative PNI combined with GGT/ALT would be an effective and practical predictor of prognosis in patients with HCC within the Milan criteria.

## Materials and methods

### Patients

All patients who underwent hepatectomy for histologically proven HCC at Shengjing Hospital between January 2012 and December 2019 were identified. The inclusion criteria were as follows: (a) tumor within the Milan criteria: single tumor ≤ 5 cm in diameter, or tumor number ≤ 3, a maximum diameter of ≤ 3 cm, (b) R0 resection achieved, (c) no macroscopic vascular or bile duct invasion, and (d) hepatectomy as initial treatment in Shengjing Hospital. The exclusion criteria were as follows: (a) history of second primary malignant tumors; (b) severe preoperative physical condition such as cardiopulmonary compromise or renal failure; (c) the patient had an acute infection within 7 days; (d) Child–Pugh C; (e) previous chemotherapy or other preoperative antitumor treatment; and (f) incomplete data. A total of 283 patients were enrolled in this study. HCC was diagnosed based on the American Association for the Study of Liver Diseases (AASLD) and the European Association for the Study of the Liver (EASL) (20, 21). A total of 283 patients completed the retrospective study. This study was approved by the Ethics Committee of Shengjing Hospital. The need for written informed consent was waived owing to the retrospective nature of the study.

### Follow up

Patients were generally followed up with serum AFP levels, liver function tests, ultrasound and dynamic contrast-enhanced CT or MRI, and serum AFP levels at 2 months postoperatively, and then once every 3 months. Disease-free survival (DFS) was defined as the interval between the date of surgery and the date of detection of recurrence. Overall survival (OS) was defined as the period between the date of hepatectomy and the date of death or the date of the last follow-up. Once recurrence was confirmed, salvage treatments were selected, including hepatectomy, MWA, or TACE.

### Statistical analysis

Categorical variables were compared using the chi-square test (or Fisher’s exact test, if necessary). Survival curves were drawn using the Kaplan–Meier method and compared using the log-rank test. Univariate and multivariate Cox regression analyses were performed to identify the prognostic factors related to OS and DFS. Time-dependent receiver operating characteristic (ROC) curve analysis was used to determine the cutoff values of PNI and GGT/ALT. The area under the ROC curve (AUC) was used to estimate and compare the prognostic predictive ability of the new scoring model with other prognostic factors. A larger AUC indicated a higher predictive power. All statistical analyses were performed using the SPSS Statistics software package (version 25.0; SPSS, Chicago, IL), and statistical significance was set at a two-tailed p < 0.05.

## Result

### Cut-off determination of PNI and GGT/ALT

PNI was defined as albumin level (g/L) + 5 × lymphocyte count (109/L). The optimal cut-off value of PNI was 48.48, sensitivity = 0.688, and specificity = 0.581, corresponding to the maximum Youden index (= 0.269) for predicting 5-year OS in ROC curve analysis. Therefore, patients were divided into the PNI-high (PNI ≥48.48) and PNI-low (PNI < 48.48) groups. There were 100 patients with low PNI and 183 with high PNI.

The optimal cut-off value of GGT/ALT was 1.65, sensitivity was 0.628, and specificity was 0.621, corresponding to the maximum Youden index of 0.249 for predicting 5-year OS in ROC curve analysis. Patients were then assigned to either the GGT/ALT-high (GGT/ALT ≥1.65; n=118) or GGT/ALT-low (GGT/ALT < 1.65; n=165) group.

### Patient characteristics

A total of 283 patients (men, 223 [78.8%]; women, 60 [24.2%]; median age, 58 years [range: 30–79 years]) were enrolled in this study; their baseline characteristics are shown in **Table 1**. The median follow-up time was 40 months (range, 2–107 months). During the follow-up period, 47 (16.6%) patients died, and 110 (38.9%) had tumor recurrence. The 1-, 3-, and 5-year overall survival (OS) rates were 94.7%, 87.3%, and 84.8%, respectively. The 1-, 3-, and 5-year DFS rates were 82.0%, 68.9%, and 64.3%, respectively.

**Table 1:** Baseline characteristics by treatment cohort.

	Total	PNI		p	GGT/ALT		p
Variables		<48.48	≥48.48		<1.65	≥1.65	
Age (≥60 years)	115 (40.6)	42 (42.0)	73 (39.9)	0.730	67 (40.6)	48 (40.7)	0.990
Sex (male)	223 (78.8)	74 (74.0)	149 (81.4)	0.144	129 (78.2)	94 (79.7)	0.764
Virus hepatitis	257 (90.8)	90 (90.0)	167 (91.3)	0.726	154 (93.3)	103 (87.3)	0.083
Tumor number (multiple)	24(8.5)	9 (9.0)	15 (8.2)	0.817	11 (6.7)	13 (11.0)	0.195
Tumor size (>3 cm)	135 (47.7)	52 (52.0)	83 (45.4)	0.285	78 (47.3)	57 (48.3)	0.864
PLT (< 100 × 10^9/L)	95(33.6)	63 (63.0)	32(17.5)	<0.001	57 (34.5)	38 (32.2)	0.681
AFP > 200 ng/mL	73 (25.8)	27 (27.0)	46 (25.1)	0.732	43 (26.1)	30 (25.4)	0.904
ALB (< 35 g/L)	23 (8.1)	23 (23.0)	0 (0)	<0.001	12 (7.3)	11 (9.3)	0.534
TBIL (> 17.1 μmol/L)	76 (26.9)	39 (39.0)	37 (20.2)	0.001	40 (24.2)	36 (30.5)	0.241
ALT ( > 40 U/L)	94 (33.2)	40 (40.0)	54 (29.5)	0.073	59 (35.8)	35 (29.7)	0.283
AST (> 40 U/L)	64 (22.6)	45 (45.0)	19 (10.4)	<0.001	38 (23.0)	26 (22.0)	0.843
PT (prolongation > 3 s)	7 (2.5)	4 (4.0)	3 (1.6)	0.411	4 (2.4)	3 (2.5)	1.000
Cirrhosis	217 (76.7)	90 (90.0)	127 (69.4)	<0.001	127 (77.0)	90 (76.3)	0.891
Portal hypertension	123 (43.5)	68 (68.0)	55 (30.1)	<0.001	67 (40.6)	56 (47.5)	0.252
Child-Pugh B	21(7.4)	17 (17.0)	4 (2.2)	<0.001	8 (4.8)	13 (11.0)	0.051
GGT(> 50 U/L)	(103.9)	56 (56.0)	72 (39.3)	0.007	40 (24.2)	88 (74.6)	<0.001

PNI, prognostic nutritional index; GGT, γ-glutamyl transferase; PLT, platelets; AFP, alpha-fetoprotein; TBIL, total bilirubin; ALB, albumin; ALT, alanine aminotransferase; AST, aspartate aminotransferase; PT, prothrombin time.

Categorical Data presented in n (%).

### Correlation between PNI or GGT/ALT and clinicopathologiccharacteristics of HCC

The clinicopathological characteristics of the patients with different PNI or GGT/ALT levels were analyzed (**Table 1**). High GGT/ALT levels were only related to GGT (> 50 U/L) (P < 0.001). However, low PNI was related not only to GGT (> 50 U/L) (P = 0.007), but also to PLT (< 100 × 10^9/L), ALB (< 35 g/L), TBIL (> 17.1 μmol/L), ALT ( > 40 U/L), AST (> 40 U/L), Cirrhosis, portal hypertension and Child-Pugh B (all p < 0.05).

### Factors associated with OS and DFS in patients with HCC

Univariate and multivariate Cox regression analyses were performed to identify the factors related to OS and DFS (**Table 2**). Univariate analyses showed that multiple tumor numbers, low PLT (< 100 × 10^9/L), decreased ALB (< 35 g/L), portal hypertension, low PNI (< 48.48), and high GGT/ALT ratio (≥ 1.65) were associated with poor OS. In multivariate analyses, multiple tumor numbers, low PNI, and high GGT/ALT remained independent prognostic factors. Univariate analysis of these parameters revealed that multiple tumor numbers, low PLT, portal hypertension, low PNI, and high GGT/ALT were associated with poor DFS, but multivariate analysis showed that only multiple tumor numbers and high GGT/ALT were independent risk factors for DFS.

**Table 2:** Prognostic factors of overall survival and recurrence-free survival.

Variables	Overall survival				Disease-free survival survival			
	Univariate	p	Multivariate	p	Univariate	p	Multivariate	p
	HR (95% CI)		HR (95% CI)		HR (95% CI)		HR (95% CI)	
Age (≥60 years)	1.29 (0.73 – 2.30)	0.378			0.74 (0.50 –1.10)	0.133		
Sex (male)	1.42 (0.63 – 3.17)	0.395			1.17 (0.72 – 1.89)	0.536		
Viral hepatitis	0.58 (0.24 – 1.36)	0.209			1.30 (0.60 – 2.80)	0.505		
Tumor number (multiple)	3.60 (1.73 – 7.51)	0.001	3.64 (1.71 – 7.75)	0.001	2.30 (1.32 – 3.98)	0.003	2.14 (1.23 – 3.73)	0.007
Tumor size (>3 cm)	1.26 (0.71 – 2.23)	0.431			1.28 (0.88 – 1.86)	0.196		
PLT (< 100 × 10^9/L)	2.40 (1.35 – 4.26)	0.003	1.43 (0.69 – 2.97)	0.340	1.53 (1.04 – 2.25)	0.033	1.13 (0.69 – 1.85)	0.633
AFP (> 200 ng/mL)	0.95 (0.49 – 1.84)	0.885			1.40 (0.94 – 2.10)	0.099		
ALB (< 35 g/L)	2.21 (1.03 – 4.73)	0.041	1.25 (0.54 – 2.91)	0.600	0.67 (0.29 – 1.53)	0.343		
TBIL (> 17.1 μmol/L)	1.73 (0.96 – 3.11)	0.070			1.10 (0.72 – 1.68)	0.656		
ALT ( > 40 U/L)	1.22 (0.68 – 2.21)	0.500			1.00 (0.67 – 1.50)	0.986		
AST (> 40 U/L)	1.66 (0.90 – 3.07)	0.106			1.07 (0.68 – 1.69)	0.759		
PT (prolongation > 3 s)	1.97 (0.48 – 8.14)	0.349			1.46 (0.54 – 3.97)	0.457		
Cirrhosis	1.52 (0.71 – 3.25)	0.281			1.05 (0.67 – 1.64)	0.843		
Portal hypertension	2.15 (1.20 – 3.86)	0.010	1.28 (0.63 – 2.61)	0.493	1.56 (1.07 – 2.27)	0.020	1.25 (0.79 – 1.98)	0.336
PNI (< 48.48)	2.84 (1.59 – 5.10)	<0.001	2.13 (1.08 – 4.21)	0.029	1.63 (1.11 – 2.37)	0.012	1.41 (0.92 – 2.16)	0.114
GGT/ALT (≥1.65)	2.47 (1.37 – 4.45)	0.003	2.34 (1.29 – 4.24)	0.005	1.74 (1.19 – 2.52)	0.004	1.65 (1.13 – 2.41)	0.010

HR, hazard ratio; PLT, platelets; AFP, alpha-fetoprotein; ALB, albumin; TBIL, total bilirubin; ALT, alanine aminotransferase; AST, aspartate aminotransferase; PT, prothrombin time; PNI, prognostic nutritional index; GGT, γ-glutamyl transferase.

Bold means there is statistically significant. Variables with P < 0.05 at univariate analysis were retained for multivariate analysis.

### Preoperative prognostic scoring model based on nutrition and inflammation

The preoperative prognostic scoring model was based on nutritional status (PNI) and liver inflammation markers (GGT/ALT). Patients with a high PNI were assigned a score of 0; otherwise, the patients were assigned a score of 1. Patients in the GGT/ALT-low (GGT/ALT ≥1.65) group were assigned a score of 0; patients in the GGT/ALT-high (GGT/ALT < 1.65) group were assigned a score of 1. Subsequently, these values were used to calculate the PNI-GGT/ALT score for each patient. The combination of the PNI and GGT/ALT scores was the summation of the two scores, and the patients were then divided into grade 1 (score 0), grade 2 (score 1), and grade 3 (score 2). Among the 283 patients in this study, 107(37.8%) were reclassified as grade 1, 134(47.3%) as grade 2, and 42(14.8%) as grade 3.

### Survival analysis

The median survival duration was 40 months (range 2–107 months). A total of 47/283 (16.6%) patients died, and 110/283 (38.9%) patients experienced tumor recurrence during the follow-up period.

**Figure 1:**
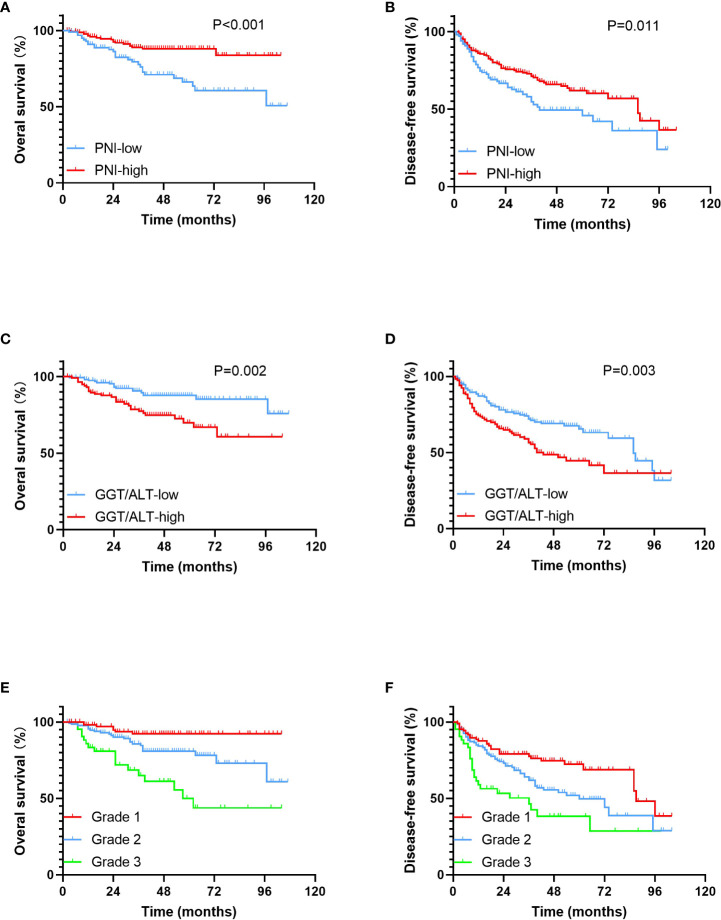
Kaplan-Meier survival curves for OS and DFS in patients within the Milan criteria HCC. **(A)**. OS rates stratified by PNI; **(B)**. DFS rates stratified by PNI; **(C)**. OS rates stratified by GGT/ALT; **(D)**. DFS rates stratified by GGT/ALT; **(E)**. OS rates stratified by PNI-GGT/ALT grade; **(F)**. DFS rates stratified by PNI-GGT/ALT grade.

Compared with the PNI-low group, the patients in the PNI-high group had markedly higher 1-, 3-, and 5-year OS rates (96.6%, 89.0%, and 88.2% vs. 90.9%, 77.8%, and 66.3%, respectively; P < 0.001) (**Figure 1A**) and DFS rates (85.5%, 71.1%, and 62.0% vs. 74.6%, 54.7%, and 45.9%, respectively; P = 0.011) (**Figure 1B**), suggesting a positive correlation between low PNI and poor OS and DFS.

Compared with the GGT/ALT-low group, the patients in the GGT/ALT-high group had markedly poorer 1-, 3-, and 5-year OS rates (90.5%, 77.5%, and 69.9% vs. 97.5%, 90.6%, and 87.7%, respectively; P = 0.002) (**Figure 1C**) and DFS rates (74.3%, 56.5%, and 44.5% vs. 87.0%, 72.1%, and 65.4%, respectively; P = 0.003) (**Figure 1D**), suggesting a positive correlation between high GGT/ALT levels and poor survival in patients with HCC.

The 1-, 3-, 5- year OS rates in the PNI-GGT/ALT grade 1 group were 98.0%, 92.2%, and 92.2%, respectively and those in the PNI-GGT/ALT grade 2 group were 95.4%, 85.7%, and 80.9%, respectively. The 1-, 3-, and 5-year OS rates in the PNI-GGT/ALT grade 3 group were 83.3%, 65.0%, and 50%, respectively (P < 0.001) (**Figure 1E**). And DFS rates in the PNI-GGT/ALT grade 1 group were 87.6%, 77.6%, and 72.3%, respectively. The 1-, 3-, and 5-year DFS rates in the PNI-GGT/ALT grade 2 group were 84.2%, 61.7%, and 49.6%, respectively. The 1-, 3-, and 5-year DFS rates in the PNI-GGT/ALT grade 3 group were 58.8%, 46.4%, and 38.3%, respectively (P < 0.001) (**Figure 1F**). Compared with the PNI-GGT/ALT grade 2 group, the PNI-GGT/ALT grade 1 group had significantly higher 1-, 3-, and 5-year OS (P = 0.013) and DFS (P = 0.013) rates. Likewise, compared with the PNI-GGT/ALT grade 3 group, the PNI-GGT/ALT grade 2 group had significantly higher 1-, 3-, and 5-year OS (P = 0.002) and DFS (P = 0.022) rates.

**Figure 2:**
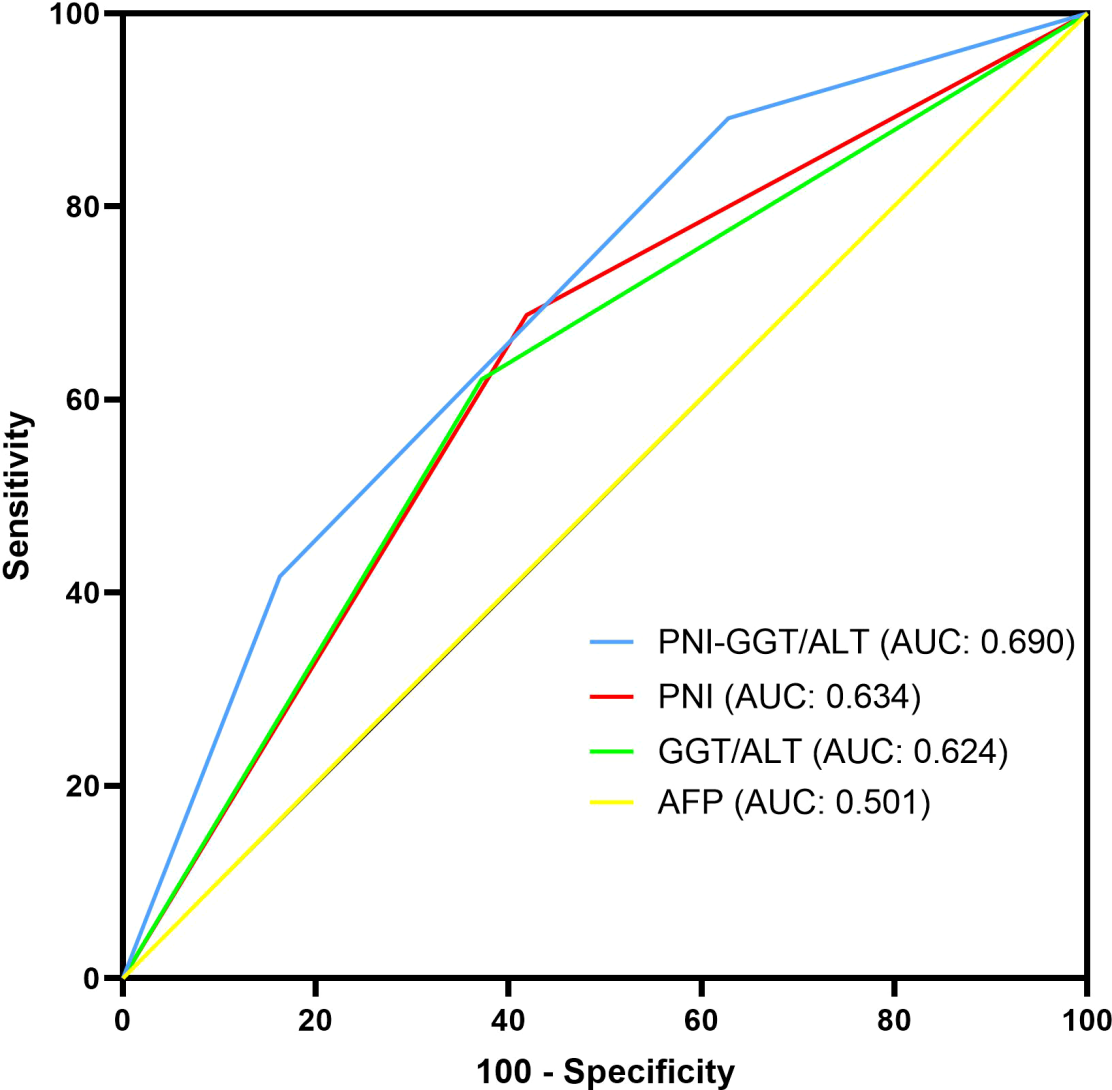
Comparison of the AUC of PNI-GGT/ALT, PNI, GGT/ ALT and AFP in predicting the 5-year OS of patients within the Milan criteria HCC.

In addition, time-dependent ROC curves were used to compare the prognostic efficacy of PNI-GGT/ALT, PNI, GGT/ALT and AFP (**Figure 2**). Our results indicated that PNI-GGT/ALT had a higher AUC value (0.690) in predicting the 5- year OS of patients within the Milan criteria HCC after curative resection in comparison with AFP (0.501), PNI (0.634) and GGT/ALT (0.624).

## Discussion

Risk factors for HCC include chronic hepatitis B and C, alcohol addiction, nonalcoholic fatty liver disease, and exposure to aflatoxin (5). Chronic inflammation is a hallmark of HCC (22), despite intrinsic differences among etiological factors for HCC. The pathogenesis of HCC is based on the perpetuation of a wound-healing response triggered by parenchymal cell death and the ensuing inflammatory reaction (23). The inflammatory environment contributes to constant cell death, compensatory regeneration and activation of non-parenchymal cells, the survival of preneoplastic hepatocytes, the generation of a fibrotic substrate, and is ultimately believed to contribute to tumorigenic progression (23, 24). The necroinflammation induces altered survival and proliferation signals, cellular stress, epigenetic modifications, chromosomal aberrations, mitochondrial alterations and senescence (24). Inflammation also leads to proliferation-associated replication stress, DNA damage and genetic instability, which can be detected before tumorigenesis (25).

In addition, changes in nutritional status can affect tumor progression and ultimately affect cancer prognosis. The liver is the central organ involved in the metabolism of nutrients, and patients with HCC are at a significantly increased risk for malnutrition. Prospective clinical studies have identified malnutrition as an independent negative prognostic factor in patients with HCC (26). Preoperative malnutrition may lead to increased postoperative infection and complications, resulting in delayed recovery (27). The predictive role of inflammation and nutrition markers in the prognosis of patients with HCC is of increasing interest.

The PNI is a combination index that reflects the nutritional status, immune function, and systemic inflammation of patients. It was first proposed to be an independent predictor of survival in patients with HCC by Pinato et al. (28) PNI was a better prognostic factor in patients with early HCC than in those with advanced stage diseases (29), and all the patients in this study were in the early stage.

The reasons why PNI can predict the prognosis of patients with HCC are as follows: As a negative acute-phase protein, albumin decreases in inflammatory states (30). Low serum albumin levels means the presence of cancer cachexia is caused by a sustained inflammatory response, either from the tumor itself or as a host reaction (31). In addition, hypoalbuminemia reflects a state of malnutrition, which would weaken cellular and humoral immunity, phagocytic functions, and other defense mechanisms in cancer patients (32). A recent study first reported that ALB acts as a tumour suppressor and plays a vital role in HCC progression, particularly in invasion and metastasis. Down-regulation of ALB promoted migration and invasion of HCC cells by increasing uPAR, matrix metalloproteinase (MMP2), and MMP9 (33). Supplementation with branched-chain amino acids can improve outcomes after cancer therapy and inhibit early relapse after hepatectomy (34). Lymphocytes play an important role in cancer surveillance (35). Accumulating evidence supported that the interactions of infiltrating immune cells with tumor components act as vital driving factors for tumor progression and therapeutic sensitivity (36, 37). As the prime anti-tumor cells, CD8^+^ T cells can secrete IFN-γ and TNFα to destroy cancerous cells once they contact tumor cells or eliminate tumor cells by releasing perforin and granzyme B through the Fas/FasL pathway (38). A large study of patients with HCC highlighted that a progressive lack of CD4^+^ T cells is closely associated with HCC progression and poor survival rates in HCC patients (39). The recruitment and activation of lymphocytes in tumor microenvironment inhibit tumor cell proliferation, migration, invasion, and chemoresistance (40). A lower lymphocyte count may indicate impairment of innate cellular immunity and a weakened defense against cancer, and ultimately enhance the malignant biological behavior of cancer cells.

Gamma-glutamyltransferase (γ-GT) is a membrane-binding enzyme that regulates the metabolism of glutathione and can exert pro-oxidative effects at the membrane surface and in the extracellular microenvironment (41, 42). However, the pro-oxidative activity of GGT may contribute to persistent oxidative stress described in cancer, modulate processes involved in tumor progression, such as cell proliferation or apoptosis, and to protective adaptation against electrophilic or alkylating compounds (42). High GGT levels are significantly associated with increased risk of esophageal, lung, laryngeal, colorectal, and stomach cancer, particularly cancer of the liver (43).

Increased GGT/ALT ratio was originally associated with worse prognosis and treatment response in chronic viral hepatitis (15, 16). Yang et al. reported that hepatitis B patients with a higher GGT/ALT ratio were more likely to develop liver cancer (44). Vascular invasion is a significant but poor prognostic factor of survival in patients with HCC (45), Luo et al. disclosed that a high GGT/ALT ratio was identified as an independent risk factor for vascular invasion in patients with HCC (46). Additionally, Ju et al. observed that a higher GGT/ALT ratio correlated with a more severe tumor burden in patients with HBV-related HCC, including tumor size, tumor capsule, and shortened survival time (18).

To the best of our knowledge, this study is the first to assess the prognostic, predictive capacity of the combined PNI and GGT/ALT in patients with HCC within the Milan criteria after hepatectomy. In this study, we found that although both PNI and GGT/ALT could predict the prognosis of patients with HCC within the Milan criteria, their low accuracy and mutual exclusion as independent predictors suggest a possible combination. Therefore, the novel prognostic score model based on PNI and GGT/ALT, the PNI-GGT/ALT score is a combination of PNI as a systemic inflammation- and nutrition-related marker, and GGT/ALT is a liver inflammation-related marker that can increase the predictive values. The new prognostic score had a higher AUC value for predicting the 5-year survival of patients with HCC within the Milan criteria after curative resection (0.690) than AFP (0.501), PNI (0.634) and GGT/ALT (0.624). According to the survival analysis, OS and DFS of patients with higher PNI-GGT/ALT score are both shorter than patients with lower score(P < 0.05), which indicated that PNI-GGT/ALT grade might be a promising stratification tool for HCC patients within Milan criteria.

Owing to the advantages of being inexpensive and easily obtained at outpatient clinics, PNI, and GGT/ALT have been extensively investigated and identified as independent prognostic factors in patients with HCC, at least to a certain extent. The PNI and GGT/ALT ratios were integrated to create a novel prognostic model that takes into account the nutritional status, systemic inflammation, and liver inflammation.

However, this study had several limitations. First, this was a retrospective study, which may have led to selection bias. Second, our study set the cutoff values of PNI and GGT/ALT to be 48.48 and 1.65, respectively, but the optimal cutoff values of PNI and GGT/ALT remain to be determined. Third, this was a single-institution study of a homogeneous population with hepatitis B virus (HBV) (88.2%) as the main risk factor for HCC. Whether these findings can be applied to Western populations, wherein hepatitis C virus, alcoholic addiction, metabolic liver disease, and other etiologies of liver disease predominate, requires further study and discussion. Hence, further large-scale, multicenter, randomized controlled trials are required to confirm these results.

In conclusion, the PNI and GGT/ALT ratios were integrated to create a novel prognostic model for patients with HCC within the Milan criteria after curative resection that considers the nutritional status, systemic inflammation, and liver inflammation.

## Data availability statement

The raw data supporting the conclusions of this article will be made available by the authors, without undue reservation.

## Ethics statement

The studies involving human participants were reviewed and approved by the ethics committee of Shengjing Hospital. Written informed consent for participation was not required for this study in accordance with the national legislation and the institutional requirements.

## Author contributions

HF and CD contributed to conception and design of the study. HF, FX, YZ, TJ, JL, RL and TZ organized the database. HF performed the statistical analysis. HF wrote the first draft of the manuscript. All authors contributed to the article and approved the submitted version.

## Funding

This work was supported by the Shenyang Science and Technology Project under Grant [number 7-230-9-16]; Liaoning Province Key Research and Development Plan Guidance Project under Grant [number 2017225032].

## Acknowledgments

Hanxin Feng wants to thank Yutong Huang for her company.

## Conflict of interest

The authors declare that the research was conducted in the absence of any commercial or financial relationships that could be construed as a potential conflict of interest.

## Publisher’s note

All claims expressed in this article are solely those of the authors and do not necessarily represent those of their affiliated organizations, or those of the publisher, the editors and the reviewers. Any product that may be evaluated in this article, or claim that may be made by its manufacturer, is not guaranteed or endorsed by the publisher.
